# Anti-Suicide Function of Nonsuicidal Self-Injury in Female Inpatient Adolescents

**DOI:** 10.3389/fpsyt.2020.00490

**Published:** 2020-06-03

**Authors:** Laura Kraus, Marc Schmid, Tina In-Albon

**Affiliations:** ^1^Clinical Child and Adolescent Psychology, University of Koblenz-Landau, Landau, Germany; ^2^Department of Child and Adolescent Psychiatry, University of Basel, Basel, Switzerland

**Keywords:** nonsuicidal self-injury disorder, adolescents, anti-suicide, suicidality, suicidal behavior disorder

## Abstract

**Background:**

There have been numerous studies investigating the relationship between nonsuicidal self-injury (NSSI) and suicidality. On the one side, NSSI is an important risk factor for suicidality, including suicidal thoughts and behaviors. On the other side, it has been suggested that one function of NSSI might be as a coping mechanism that can help individuals in the short term avoid suicide. The present study investigated the relationship between suicidality and NSSI in female inpatient adolescents, focusing on NSSI as an anti-suicide strategy.

**Methods:**

Subjects were 56 female adolescents, aged 12–18 years (*M* = 15.95 years, *SD* = 1.27), recruited from different inpatient child and adolescent psychiatric units. All participants fulfilled the *DSM-5* research criteria for nonsuicidal self-injury disorder (NSSI-D). To assess suicidality, NSSI-D, and current and past diagnoses, a structured clinical interview was conducted.

**Results:**

NSSI as a short-term coping strategy for avoiding suicide was indicated by one third (32.1%) of the participants. Before participants engaged in NSSI, the anti-suicide function was reported more frequently than reducing interpersonal problems (*d* = -.59). Getting relief from negative emotions and inducing positive feelings were reported at the same frequency as avoiding suicide before NSSI. Participants engaging in NSSI to avoid suicide and those reporting other functions did not significantly differ regarding other NSSI characteristics, suicidality, or psychopathology. Results indicate that the anti-suicide function significantly predicts the duration of current suicidal ideation (β = .557).

**Conclusion:**

This study provides preliminary support for the idea that NSSI is frequently used by female adolescents with NSSI-D to avoid suicide. Given the high co-occurrence of NSSI and suicidality, our results underline the importance of clinical assessment of suicidality and several NSSI functions, including the anti-suicide function, in adolescents with NSSI.

## Introduction

According to the *Diagnostic and Statistical Manual of Mental Disorders* [5th ed.; (1)], nonsuicidal self-injury (NSSI) is defined as repetitive (at least 5 days in 1 year), socially unaccepted acts of intentional self-inflicted damage to one’s own body tissue without suicidal intent. Previous research has shown that NSSI is highly prevalent during adolescence, affecting around 8% of adolescents in a German-speaking sample within the last 6 months (2), with even higher rates (around 50%) in hospitalized adolescents (3).

In addition to the high prevalence rates, of special concern is that NSSI is a major risk factor for suicidal thoughts and behaviors [(4); see also (5), for a systematic review and meta-analysis]. Although they often co-occur, as adolescents with NSSI also report frequent suicidal thoughts (6, 7), NSSI and suicidal behavior are discrete entities. In the *DSM-5* (1), research criteria for NSSI and for suicidal behavior disorder (SBD) have been included in Section III. SBD is characterized by a suicide attempt in the past 24 months, not including NSSI or suicidal ideation. Previous research has examined the association between NSSI and suicidality, focusing on both distinction and commonality. A systematic review concluded that most studies distinguished NSSI from suicidal behavior in relation to the intention to die (8). In addition, methods and injuries of NSSI are often less severe and usually the damage is not life threatening. NSSI and suicide also differ in the frequency of the act, as NSSI often occurs daily (9). Victor and Klonsky (10) conducted a meta-analysis on correlates of suicide attempts among individuals engaging in NSSI. The strongest predictors of suicide attempt history were self-injury frequency, number of methods, and hopelessness; moderate predictors were borderline personality disorder (BPD), impulsivity, posttraumatic stress disorder (PTSD), cutting as a NSSI method, and depression.

There are different theoretical models that have been proposed to explain the link between NSSI and suicidal behavior. Hamza et al. (11) introduced an integrated model, considering previous theories such as the gateway theory [(12); continuum of NSSI to suicidal behavior as several studies found that NSSI took place before suicidal behavior started, e.g., (13, 14)], the third variable theory (that the association between NSSI and suicidal behavior is spurious, and that a third variable, e.g., BPD, accounts for the co-occurrence; (15), and Joiner’s (16) interpersonal theory of suicide (that attempting suicide requires the desire and capability for suicide and that repeated experience with painful acts, including NSSI or suicidal behavior, leads to a higher acquired capability for lethal self-injury, i.e., an habituation process) as none of these theories alone is clearly supported. To date, only one theoretical framework, the anti-suicide model (17), has considered the anti-suicide function of NSSI, suggesting NSSI could be protective against suicide. However, there is also some evidence that engaging in NSSI to avoid suicide might be a risk factor for suicidal behavior. Therefore, further research is needed to clarify the link between the anti-suicide function of NSSI and suicidality.

It has been established that NSSI is motivated by a broad variety of different functions that can be categorized as intrapersonal/self-regulating or interpersonal/social. A random effects meta-analysis of the prevalence of different functions found that intrapersonal functions, with a prevalence of 66%–81%, and especially emotion regulation, with 63%–78%, were more prevalent than interpersonal functions with 33%–56% (18). Frequently, NSSI serves multiple functions simultaneously (6), which in turn maintain NSSI, and thus different underlying functions of NSSI may have different needs regarding interventions. Although emotion regulation is a prevalent function, it should be noted that not every individual engages in NSSI for this reason. Therefore, each individual functions should receive attention and should be assessed in any case (18, 19). Several studies found significant associations between intrapersonal NSSI functions and suicidal ideation and attempts (4, 20). Similarly, Nock and Prinstein (21) found in a clinical sample of adolescents with NSSI a significant correlation between a history of suicide attempts and automatic negative reinforcement (e.g., “to stop bad feelings”). This result was replicated in latent class analyses by Klonsky and Olino (22) and Case et al. (19), who found that intrapersonal reasons for NSSI went with high levels of suicidal ideation and behaviors. As NSSI serves multiple functions, it is not surprising that different functions (function accumulation) have been associated with higher risk for suicidal ideation and behaviors (23). Adolescents who attempted suicide have reported that they did so to escape negative experiences (24), suggesting a preliminary functional similarity between NSSI and suicide attempts.

As described above with the theoretical anti-suicide model of Suyemoto (17), it has been further suggested that NSSI functions as one of several “coping” mechanisms for resisting urges to engage in suicide. The anti-suicide function of NSSI, defined as self-injury to replace, compromise with, or avoid suicide (25), is considered an intrapersonal function (26). Previous studies emphasized the association between the anti-suicide function of NSSI and suicidal behavior (4, 20, 27, 28) and suicidal ideation in adolescents and young adults engaging in NSSI (28). There is some evidence that NSSI could be a protective factor against suicide, used as a way of dealing with suicidal behaviors (17, 29, 30). In Suyemoto’s (17) anti-suicide model, rooted in psychoanalytic theory, NSSI represents a compromise and is therefore used instead of suicide. NSSI might therefore be an active coping mechanism (17) and might be a way to express suicidal thoughts without risking death for some individuals (25). Similarly, several authors hypothesized that individuals struggling with suicidal ideation may engage in NSSI as a short-term way to relieve suicidal thoughts, which may lead to greater NSSI engagement, explained by the negative reinforcement (27, 28). Nevertheless, some authors have argued that individuals who engage in NSSI to avoid suicide might be at higher risk for suicidal behaviors (20, 27). The results of Burke et al. (27) indicate that the anti-suicide function of NSSI and depressive symptoms are the two most important predictors of suicidal ideation and planning. Moreover, participants who reported higher identification with NSSI to avoid suicide were more likely to report lifetime suicide attempts than those without NSSI (27). These results indicate that it is relevant to investigate various functions of NSSI, to understand the risks associated with NSSI (31).

Brausch and Muehlenkamp (4) investigated the extent of perceived effectiveness in achieving a desired function (e.g., to avoid suicide). The authors reported that the perceived effectiveness of the anti-suicide function was a significant predictor of lifetime suicidal ideation and suicide attempts as well as intensity of suicidal ideation in adults with NSSI. Examining first-hand accounts of the reasons for self-harm (defined as nonfatal acts of intentional self-injury or self-poisoning) other than intent to die, a systematic review found that the anti-suicide function was reported in only 15% of quantitative and 7% of qualitative studies (32), indicating that the function of NSSI to avoid suicide has not been assessed regularly.

Thus, there is preliminary evidence that engaging in NSSI to avoid suicide could be protective against suicidal behavior, but numerous studies have also emphasized the risk factor for suicidality. Therefore, more studies investigating the anti-suicide function are necessary, as previous studies that examined this function had several limitations. For example, some studies investigated the anti-suicide function using only self-report questionnaires (27, 28) or a Web-based survey (20) instead of using structured clinical interviews to gain more detailed information. Generalizability and comparability of previous findings could be restricted because they involved different samples. So far, only one study has included adolescents and young adult patients receiving treatment for NSSI (28); others investigated university students (20, 27). To date, little research has specifically investigated how engaging in NSSI to avoid suicide is associated with other NSSI characteristics and suicidality in adolescents with NSSI-D.

Therefore, the present study investigated the relationship between suicidality and NSSI in inpatient adolescents with NSSI disorder (NSSI-D), focusing on various NSSI functions including an anti-suicide strategy. The aim of the study was first to investigate the anti-suicide function in adolescents with NSSI-D, using a structured clinical interview. According to the *DSM-5* research criteria for NSSI-D (1), individuals engage in NSSI with the expectation of relieving negative feelings or cognitions, resolving interpersonal difficulties, or inducing a positive feeling state (criterion B). It should be noted that the relief or response takes place during or after NSSI. Therefore, we investigated this criterion. Using an exploratory approach, we examined these expectations before, during, and after engaging in NSSI (like a time course). Suyemoto (17) hypothesized that individuals with major depression are more likely to engage in NSSI to avoid suicide. Furthermore, as already mentioned, some studies emphasized that NSSI to avoid suicide might be a risk factor for suicidality. NSSI to avoid suicide showed the strongest correlation to suicide attempts among university students and was more frequently reported in individuals who had attempted suicide than those who reported suicidal ideation (20). Therefore, we compared participants who engaged in NSSI to avoid suicide with participants reporting other functions of NSSI regarding suicidality, NSSI characteristics, and psychopathology. Second, we explored predictors of current suicidal ideation and lifetime suicide attempts, replicating previous findings that the perceived effectiveness of the anti-suicide function was a significant predictor of lifetime suicidal ideation and suicide attempts as well as the intensity of suicidal ideation in adults with NSSI (4). Thus, we investigated whether the anti-suicide function is a predictor for a wide spectrum of suicidal behaviors in female adolescents, considering suicidal ideation (intensity and duration) and suicide attempts.

In summary, we addressed the following research questions:

1. How frequently do adolescents engaging in NSSI indicate a desire to avoid a suicide attempt compared to other functions indicated in the *DSM-5* research criteria for NSSI-D?
2. Do adolescents engaging in NSSI to avoid a suicide attempt differ from those reporting other NSSI functions with regard to suicidality, NSSI characteristics, and psychopathology?
3. Does the anti-suicide function predict current suicidal ideation (intensity and duration) and lifetime suicide attempts?

## Materials and Methods

### Participants

Participants were recruited from several inpatient child and adolescent psychiatric units in Switzerland and Germany. The total sample comprised 56 female adolescents, aged 12–18 years (*M* = 15.95 years, *SD* = 1.27), all fulfilling the *DSM-5* (1) research criteria for NSSI-D. Most had Swiss or German nationality, except for one Thai and one Polish citizen. The inpatient clinics were responsible for the recruitment. Therefore, we have no access to the demographic and clinical characteristics of patients excluded by the clinics. Our predefined exclusion criteria were current or past psychosis, schizophrenic symptoms, and acute substance abuse. To eliminate further impact on NSSI, participants with BPD were not included in the following analyses. In the sample, 60.1% (*n* = 34) had attempted suicide in the past 2 years and therefore fulfilled *DSM-5* (1) research criteria for SBD. Therefore, 40% (*n* = 22) of participants met the criteria for NSSI-D without meeting criteria for SBD. To compare individuals regarding suicidality, NSSI characteristics, and psychopathology, two groups were formed on the basis of reported NSSI functions, which were assessed dimensionally using a 4-point Likert scale. NSSI as a short-term coping strategy for avoiding suicide was indicated by one third (32.1%, *n* = 18) of all participants. It should be noted that these participants also reported functions other than anti-suicide. We designated these participants as the NSSI anti-suicide function (NSSI-AF) group. Functions other than suicide avoidance were reported by 68.9% of the adolescents (*n* = 38). We designated these participants as the other function of NSSI (NSSI-OF) group. Therefore, individuals engaging in NSSI to avoid suicide and for other reasons (NSSI-AF group) were compared with those not reporting NSSI to avoid suicide (NSSI-OF group). Participants reported an average of 2.38 (*SD* = 1.36, range = 0–5) current axis I mental disorders other than NSSI-D or SBD. Three adolescents (5.4%) only met the criteria for NSSI-D and/or SBD. Using a clinical interview, in both groups we found the most frequent mental disorders were major depression (83.3% in NSSI-AF, 65.8% in NSSI-OF), social phobia (22.2% in NSSI-AF, 47.4% in NSSI-OF), specific phobia (11.1% in NSSI-AF, 18.42% in NSSI-OF), and PTSD (11.1% in NSSI-AF, 13.6% in NSSI-OF). According to Fisher’s exact test, the groups did not differ significantly regarding the most frequently reported diagnoses. Furthermore, there were no significant differences between NSSI-AF and NSSI-OF groups with regard to age (NSSI-AF: *M* = 16.12 years, *SD* = 1.56 vs. NSSI-OF: *M* = 15.86 years, *SD* = 1.13, *p* = .547), years of education (NSSI-AF: *M* = 9.19, *SD* = .91 vs. NSSI-OF: *M* = 9.20, *SD* = 1.19*, p* = .991), or number of diagnoses other than NSSI and SBD (NSSI-AF: *M* = 2.44, *SD* = 1.29 vs. NSSI-OF: *M* = 2.34, *SD* = 1.40*, p* = .679).

### Measures

#### Structured Clinical Interviews

To assess current and past *DSM-IV-TR* (33) axis I disorders, the Diagnostic Interview for Mental Disorders in Children and Adolescents (Kinder-DIPS; (34), a structured interview that follows the *DSM-IV-TR*, was conducted. The Kinder-DIPS assesses the most frequent mental disorders in childhood and adolescence (all anxiety disorders, major depression, dysthymia, eating disorders, sleeping disorders, attention deficit hyperactivity disorder, and conduct disorder, as well as substance use disorders from the adult DIPS). The Kinder-DIPS is a reliable and valid structured interview (35) with high acceptance by children, parents, and interviewers (36). In addition, the interview includes criteria for SBD and NSSI, which were published in Section III of the *DSM-5* (1). To determine the presence or absence of each symptom, the interview includes reformulated questions. Included in the NSSI section, frequency of the NSSI functions (including the anti-suicide function) is assessed before, during, and after engaging in NSSI. The functions are assessed using a 4-point Likert scale of 0 (*never/seldom*) to 3 (*very often*). The clinical distress related to NSSI is measured on a 4-point Likert scale, ranging from 0 (*not at all*) to 3 (*very high*). Included in the SBD section, the intensity of current suicidal ideation is assessed using a 5-point Likert scale of 0 (*not intensive*) to 4 (*extremely intensive*). The duration of current suicidal ideation is measured using the following scale: 1 (*1–60 s*), 2 (*2–15 min*), 3 (*16–60 min*), 4 (*less than a day*), 5 (*1–2 days*), and 6 (*more than 2 days*). Interrater reliability estimates for the diagnosis of NSSI were very good (κ = 0.90). To assess BPD, the Structured Clinical Interview for DSM-IV axis II disorders (SCID-II; (37) was conducted. Before conducting the interviews, all interviewers received an intensive standardized training.

#### FASM

The Functional Assessment of Self-Mutilation [FASM; (38)] is a self-report measure that assesses NSSI during the past 12 months. It consists of a checklist of 11 different methods of NSSI (e.g., cutting or hitting oneself) and assesses both frequency and potential received medical treatment. Furthermore, it includes 22 items assessing several functions of NSSI. Items are measured on a 4-point Likert scale, ranging from 0 (*never*) to 3 (*often*). Studies have confirmed the validity of the FASM (38) and have also shown adequate levels of internal consistency (3, 39).

#### YSR

The Youth Self Report [YSR; (40, 41)] is a self-report questionnaire measuring psychopathology in adolescents. Emotional and behavioral problems during the last 6 months are rated with a 4-point Likert scale ranging from 0 (*not true*) to 3 (*often true*). The YSR consists of 118 items that are divided between the following eight scales: somatic complaints, withdrawn, anxious/depressed, attention problems, social problems, thought problems, aggressive behavior, and delinquent behavior. Additionally, two global scales—internalized problems and externalized problems—as well as total problems can be calculated. The internal consistency of the YSR has shown to be adequate for most dimensions (i.e., anxious/depressed, aggressive behavior, and internalizing/externalizing). Furthermore, studies have shown adequate discriminant and convergent validity (42, 43).

#### BDI-II

The Beck Depression Inventory-II [BDI-II; (44)] measures depression severity and consists of 21 items that focus on the last 2 weeks. The BDI-II is a reliable (Cronbach’s α = .92 to.94) and valid measure for assessing the severity of depressive symptoms for adolescents and adults (45, 46).

### Procedure

All participants and their parents were informed about the study and provided their informed written consent in accordance with the Declaration of Helsinki. The local ethics committee approved the study. Adolescents were paid 40 Swiss francs for study participation.

### Statistical Analysis

All statistical analyses were performed using Statistical Package for the Social Sciences (SPSS) version 25.0. Descriptive statistics were calculated for both the NSSI-AF and NSSI-OF groups. Analyses of variance (ANOVAs) or nonparametric Mann–Whitney *U* or Friedman tests were used to analyze continuous variables. Pearson’s chi-square or Fisher’s exact test, including Yates’s correction for continuity, was used to investigate dichotomous variables. In addition, effect sizes (Cohen’s *d, r*, or phi) were calculated to analyze significant differences. Significance levels were set at α = 0.05. To correct for multiple testing, each *p* value was adjusted according to the Bonferroni–Holm method. The Shapiro–Wilk test and histograms were used to determine normal distribution. To analyze associations between suicidality, NSSI characteristics, depressive symptoms, and the anti-suicide function, different coefficients were calculated: Spearman correlation (two continuous variables), phi (two dichotomous variables), and point-biserial correlation (dichotomous–continuous association). Logistic regressions were performed to analyze significant predictors of current suicidal thoughts and lifetime suicide attempts (dichotomous dependent variables), using forward selection and likelihood ratio statistics. *R*² (Nagelkerke) was calculated to estimate total effect size. Multiple linear regressions were conducted to predict each intensity and duration of current suicidal ideation. Assumptions, including multicollinearity, homoscedasticity, and autocorrelation, were checked.

## Results

### Functions of NSSI

**Figure 1:**
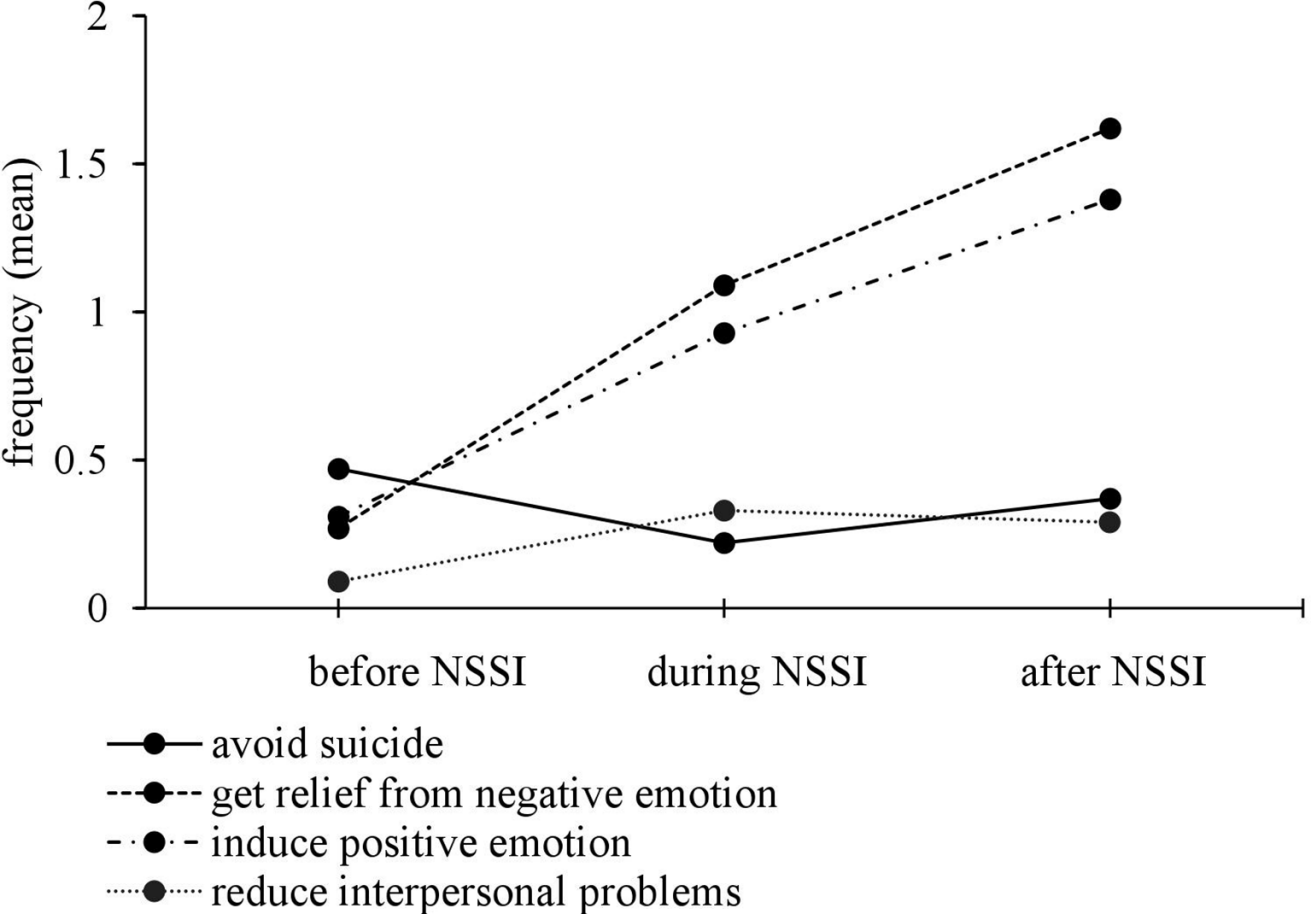
Mean frequency of the four most reported functions before, during, and after nonsuicidal self-injury.

To address the first research question on how many adolescents with NSSI-D have engaged in NSSI to avoid suicide in contrast to other functions, and how frequently, several analyses were performed. First, we examined the frequency distribution of functions. Assessing functions of NSSI according to diagnostic criterion B of NSSI-D, the most common functions were to get relief from a negative emotion (*n* = 49, 87.5%), to induce a positive emotional state (*n* = 40, 71.4%), to avoid a suicide attempt (*n* = 18, 32.1%), and to reduce interpersonal problems (*n* = 11, 19.6%). Second, regarding reported functions, we determined group differences (especially the anti-suicide function in contrast to other functions). **Figure 1** shows the mean frequency of the four most reported functions before, during, and after NSSI. As can be seen in the figure, higher differences in frequency between functions were shown during and after NSSI compared to before NSSI. Focusing on differences between reported frequency before and after NSSI, getting relief from negative emotions and inducing a positive emotional state show an increasing trend, and the anti-suicide function tends to decrease. Furthermore, ANOVAs and *post hoc* tests, including Bonferroni correction, were calculated in each case. **Table 1** presents an overview of means, standard deviation, and results of repeated ANOVAs. The results of *post hoc* tests indicate that NSSI was significantly more frequently reported as a means to avoid suicide than to reduce interpersonal problems before engaging in NSSI (*p* < .05, *d* = -.59). Before engaging in NSSI, getting relief from negative emotions and inducing positive feelings were reported at the same frequency as avoiding suicide, *p* = .226 and *p* = .175. Getting relief from a negative emotion and inducing a positive emotional state were more frequently reported than the anti-suicide function during and after NSSI, *p* < .01 in each case (*d* = .73 to .96). There were no statistically significant differences concerning NSSI as a means to avoid suicide and to reduce interpersonal problems during and after engaging in NSSI, *p* = 1.00.

**Table 1:** Means, standard deviations, and results of reported NSSI functions.

Period	NE *M* (*SD*)	PE *M* (*SD*)	IP *M* (*SD*)	AS *M* (*SD*)	*F*	Partial η²
Before NSSI	0.27 (.71)	0.31 (.79)	0.09 (.48)	0.47 (.82)	4.349*	.085
During NSSI	1.09 (1.19)	0.93 (1.20)	1.09 (1.19)	0.22 (.72)	11.171^a,^***	.195
After NSSI	1.62 (1.24)	1.38 (1.30)	0.29 (.73)	0.37 (.95)	22.388***	.332

NSSI, Nonsuicidal self-injury; NE, get relief from negative emotions; PE, create positive emotional state; IP, reduce interpersonal problems; AS, avoid suicide.

a Greenhouse–Geisser corrected.

*p < .05. ***p < .001.

### Individual Course of NSSI to Avoid Suicide

As described above, NSSI as a short-term coping mechanism for avoiding suicide was indicated by one third (32.1%) of the participants. Therefore, we focused on this subgroup of adolescents (*n* = 18). Most of these adolescents (83.3% *n* = 16) reported having thoughts about avoiding suicide before engaging in NSSI. In all, 27.8% (*n* = 5) of the participants reported thoughts about avoiding suicide during NSSI and 38.9% (*n* = 7) after NSSI. Participants reported thinking about avoiding suicide more often before (*M* = 1.28, *SD* = .89, range = 0–3) than during (*M* = 0.61, *SD* = 1.09, range = 0–3) or after (*M* = 1.00, *SD* = 1.37, range = 0–3) NSSI. A Friedman test was performed to deal with nonnormal distribution. Reported frequency of thoughts about avoiding suicide differed significantly before, during, and after NSSI, χ^2^(2) = 12.90, *p* < .01. Post hoc tests, using pairwise comparisons, indicated a significant difference in the anti-suicide function before and during NSSI with a large effect, *z* = 2.833, *p* < .05, *r* = .67. There were no significant differences in the anti-suicide function before and after NSSI, *z* = 2.167, *p* = .091, *r* = .51, as well as during and after NSSI, *z* = -.667, *p* = 1.00, *r* = -.16. In summary, frequency of the expectation of NSSI as an anti-suicide function seems to be highest before actually engaging in NSSI.

### Onset of Suicidality and NSSI

The mean age of suicidal thoughts onset in adolescents with NSSI-D was 12.77 years (*SD* = 2.05) and first suicide attempts were reported at the mean age of 14.50 years (*SD* = 1.26). The mean age of onset of NSSI was 13.24 years (*SD* = 2.22). To investigate differences in age of onset, a Friedman test was performed. Differences in the age of onset were statistically significant, χ²(2) = 15.45, *p* < .001. Post hoc tests, using pairwise comparisons, indicated that NSSI and suicidal ideation did occur significantly before first suicide attempts, *z* = .917, *p* < .05, *r* = .18. There were no age differences between onset of NSSI and suicidal ideation, *z* = .000, *p* = .1.00, indicating NSSI and suicidal ideation occurred around the same age.

### Comparison of NSSI Groups Based on Function

#### Suicidality and NSSI

##### Suicidality

In the present sample, current suicidal ideations were reported by 66%, lifetime suicide ideations by almost all of the adolescents with NSSI-D (96.3%), and lifetime suicide attempts by 70.9%. According to the *DSM-5* (APA, 2013) research criteria, adolescents both with and without NSSI as an anti-suicide function met criteria for SBD (NSSI-AF: 72.22% and NSSI-OF: 55.26%). As shown in **Table 2**, there were no significant group differences between adolescents with and without NSSI as an anti-suicide function regarding suicidal ideation and suicide attempts. Adolescents with current suicidal thoughts reported “rather intense” to “highly intense” thoughts (*M* = 2.47, *SD* = 0.96). It should be noted that there seems to be a trend that the NSSI-AF group stated longer duration of suicidal ideation (*M* = 4.18, *SD* = 1.54 vs. *M* = 3.07, *SD* = 2.16) than the NSSI-OF group that just missed statistical significance (*p* = .05, *r* = .36).

**Table 2:** Means and standard deviations of suicidality and NSSI characteristics in the NSSI-AF (*n* = 18) and NSSI-OF (*n* = 38) groups.

Characteristic	NSSI-AF	NSSI-OF	χ²	*p*	φ
	%	%			
Suicidality					
Actual SI	76.5	58.8	1.22	.270	.27
Lifetime SA	83.3	65.7	2.00	.157	.16
Lifetime SI	100.0	94.3	.95	.329	.33
	*M* (*SD*)	*M* (*SD*)	*U*	*p*	*r*
SI intensity	2.62 (.87)	2.53 (.96)	119.00	.552	.11
SI duration	4.18 (1.54)	3.07 (2.16)	52.50	.050	.36
No. of SAs	3.86 (3.11)	2.24 (1.73)	108.00	.119	.25
NSSI					
Frequency	43.23 (32.37)	33.40 (37.18)	150.00	.232	.45
No. of methods	5.11 (2.85)	3.89 (1.95)	239.00	.069	.24

AF designates participants who reported nonsuicidal self-injury (NSSI) served as an anti-suicide function; OF designates participants who reported other functions of NSSI; SA, suicide attempt; SI, suicidal ideation.

### NSSI

Participants reported an average of 41.23 (*SD* = 47.75) NSSI occasions in the past 6 months, ranging from 4 to 180 times. Furthermore, the average number of reported methods was 4.32 (*SD* = 2.30). Descriptive analyses showed the tendency of more frequent NSSI (*M* = 43.23, *SD* = 21.37 vs. *M* = 33.40, *SD* =37.18) and higher number of methods (*M* = 5.11, *SD* = 2.85 vs. *M* = 3.89, *SD* = 1.95) in adolescents using NSSI to avoid suicide than those using NSSI for other reasons, but no statistically significant differences (see **Table 2**). The adolescents reported a high level of clinical distress associated with NSSI (*M* = 1.96, *SD* = .92), with no significant group difference. In summary, there were no statistically significant group differences between the NSSI-AF and the NSSI-OF group in NSSI frequency, number of methods, or level of clinical distress.

#### Psychopathology

When examining psychopathology among female adolescents with NSSI-D, participants reported in the YSR higher levels of internalizing (*M* = 33.04, *SD* = 9.77) than externalizing (*M* = 16.89, *SD* = 9.11) problems, *t*(50) = - 10.346, *p* < .001, *r* = .83. As shown in **Table 3**, there was a nonsignificant trend (*p* = .051, *r* = .40) of higher levels of YSR total score in adolescents reporting they engaged in NSSI to avoid suicide (*M* = 114.87, *SD* = 30.16) than those reporting other functions (*M* = 99.79, *SD* = 23.63). There were no significant differences between NSSI-AF and NSSI-OF groups with regard to internalizing and externalizing problems or depressive symptoms in the BDI.

**Table 3:** Means and standard deviations of psychopathology in the NSSI-AF (*n* = 18) and NSSI-OF (*n* = 38) groups.

Psychopathology	NSSI-AF *M* (*SD*)	NSSI-OF *M* (*SD*)	*U*	*p*	*r*
YSR int	32.78 (10.30)	33.15 (9.67)	279.50	.992	.15
YSR ext	20.16 (9.86)	15.38 (8.47)	197.00	.092	.38
YSR total	114.87 (20.16)	99.79 (23.63)	184.00	.051	.40
BDI-II	33.50 (11.85)	34.16 (12.06)	262.00	.715	.19

AF designates participants who reported nonsuicidal self-injury (NSSI) served as an anti-suicide function; OF designates participants who reported other functions of NSSI; YSR, Youth Self Report (ext, externalizing problems; int, internalizing problems; total, total score); BDI-II, Beck Depression Inventory-II; U, Mann–Whitney U test.

### Logistic Regression

Correlations between anti-suicide function, NSSI characteristics, and suicidality are presented in **Table 4**. The anti-suicide function was significantly related to the duration of suicidal ideation, *r_s_* = .418, *p* < .05, but not to NSSI characteristics or occurrence of lifetime suicide attempts or suicidal ideation. Regarding NSSI characteristics, higher NSSI frequency was associated with greater use of multiple methods and vice versa, *r_s_* = .333, *p* < .05. NSSI frequency (*r_s_* = -.415, *p* < .05) and number of methods (*r_s_* = -.433, *p* < .05) were negatively related to intensity of actual suicidal ideation. Number of methods was significantly associated with the duration of suicidal ideation, *r_s_* = .430, *p* < .05. Depressive symptoms were related to the occurrence of actual suicidal ideation, *r_b_* = .446, *p* < .01, but not to intensity and duration of current suicidal ideation. Age was not correlated with NSSI characteristics or suicidality.

**Table 4:** Correlations between anti-suicide function, NSSI characteristics, suicidality, and depressive symptoms.

	1	2	3	4	5	6	7	8	9	10	11
1. Frequency	—										
2. No. of methods	.333*	—									
3. BDI-II	-.00	.095	—								
4. Actual SI	-.242	.073	.446**	—							
5. Lifetime SA	.185	.270*	.038	.136	—						
6. Lifetime SI	.156	.119	.089	.142	.087	—					
7. SI duration	-.015	.430*	-.020	c	.462**	.051	—				
8. SI intensity	-.415*	-.433*	.010	c	.056	-1.40	.213	—			
9. Anti-suicide	-.200	.196	-.123	.170	.182	.077	.418*	.136	—		
10. Clinical distress	.044	-.086	-.027	-.139	.103	.022	-.016	.458**	-.021	—	
11. Age	.190	.280	-.275	-.233	.014	.072	.091	.041	.025	.048	—

BDI-II, Beck Depression Inventory-II; c, actual suicidal ideation serves as a constant, so association cannot be calculated; NSSI, nonsuicidal self-injury; SA, suicide attempt; SI, suicidal ideation; Spearman correlation was used to analyze two continuous variables, phi was used to measure association between dichotomous variables, and point-biserial correlation was used for dichotomous-continuous association.

*p < .05. **p < .01.

To investigate if the anti-suicide function is a predictor for current suicidal ideation and lifetime suicide attempts, we performed several multiple and logistic regressions. Age was omitted as a control variable because it is not significantly related to other included variables. We first conducted two logistic regressions to analyze prediction of occurrence of current suicidal ideation and suicide attempts. After all predictors were entered, using stepwise forward selection and likelihood ratio statistics, the final models were significant for suicide attempts, χ²(1) = 5.477, *p* < .05, *R*² = .216, and suicidal ideation, χ²(1) = 6.925, *p* < .05, *R*² = .243. Results indicate that a higher number of NSSI methods significantly predicts lifetime suicide attempts, *b* = .59, *SE* = .30, odds ratio (OR) = 1.81, 95% confidence interval (CI) [1.01, 3.24], *p* < .05. Depressive symptoms, NSSI frequency, clinical distress related to NSSI, and anti-suicide function were removed from the model predicting suicide attempts. Higher depressive symptom levels significantly predict occurrence of current suicidal ideation, *b* = .09, *SE* = .040, OR = 1.09, 95% CI [1.01, 1.18], *p* < .05. NSSI characteristics and anti-suicide function do not significantly predict occurrence of current suicidal ideation.

Multiple regressions were performed to predict intensity and duration of current suicidal ideation. The model was successful in predicting duration of suicidal ideation, *F*(1,16) = 15.579, *p* < .05, *R*^2^ = .310, and results indicate that the anti-suicide function significantly predicts the duration of current suicidal ideation, β = .557, *t* = 2.680, *p* < .05; thus, higher frequency of expectation to avoid suicide leads to longer duration of current suicidal ideation. Depressive symptoms, NSSI frequency, number of methods, and clinical distress related to NSSI were not significant predictors. Finally, prediction of current intensity of suicidal ideation was analyzed. The regression model reached statistical significance, *F*(1,22) = 4.530, *p* < .05, *R*^2^ = .171. NSSI frequency in the past 6 months significantly predicts intensity of current suicidal ideation, β = -.413, *t* = -2.128, *p* < .05. The following variables were removed from the model: depressive symptoms, anti-suicide function, number of methods, and clinical distress related to NSSI.

## Discussion

The aim of the present study was to examine the anti-suicide function of NSSI in female adolescent inpatients with NSSI-D. The current study is the first to compare adolescents with NSSI-D who used NSSI also to avoid suicide with those reporting other functions. We first examined the frequency of NSSI as a means to avoid suicide in contrast to other reported functions indicated in the research criteria for NSSI-D of the *DSM-5* (1). In accordance with previous studies (18), the most highly endorsed NSSI function was intrapersonal, especially to get relief from negative emotions (87.5%). In the present study, one third of female adolescents reported engaging in NSSI to avoid suicide. The anti-suicide function of NSSI has been shown in an exploratory factor analysis to be an intrapersonal function (26) and therefore a way to regulate aversive experienced emotions.

Comparisons with previous studies on functions are difficult as most studies (4, 26) reported descriptive statistics (especially means) of several functions. Examining differences in reported functions, we found that avoiding suicide was more frequently reported than expecting to reduce interpersonal problems before engaging in NSSI. Interestingly, avoiding suicide was confirmed at the same frequency as getting relief from negative emotions and inducing a positive emotional state before engaging in NSSI. In addition, focusing on individuals engaging in NSSI to avoid suicide, frequency of the expectation that NSSI would serve an anti-suicide function seems to have been highest before individuals actually engaged in NSSI and tended to decrease while conducting NSSI. Our results therefore support the assumption that individuals may experience NSSI as a short-term coping strategy for relief from suicidal ideation and the urge to commit suicide. In summary, results indicate that the anti-suicide function is as frequent as other functions before engaging in NSSI and therefore should be assessed in each patient with NSSI.

Different functions of NSSI may have different needs regarding interventions. Therefore, intervention modules could be better individualized after identifying each patient’s specific functions. A review has shown that all efficacious NSSI treatments include individual skills training as a common element, such as emotion regulation or problem-solving strategies (47). Although skills training is an effective treatment component, practicing skills is especially difficult for patients with overwhelming emotions; such emotions may leave them unable to use these skills (48). It is therefore possible that adolescents engaging in NSSI instead of committing suicide may experience very intense emotions and, in some cases, are then unable to use coping skills. It has to be investigated if the effectiveness of coping skills is different for the various NSSI functions. Moreover, it has to be considered that the anti-suicide function represents a short-term compromise to avoid total destruction. Therefore, both the anti-suicide function and the current suicide risk need to be carefully and regularly monitored in patients with NSSI.

We further compared adolescents who engage in NSSI to avoid suicide with participants reporting other functions of NSSI. In adolescents reporting the anti-suicide function, there was a trend toward longer suicidal ideation duration, higher psychopathology, higher NSSI frequency, and higher number of NSSI methods. Though, these group differences were not significant. The failure to detect significant group differences in our study might be explained by differences in the group sizes and should be further examined with larger sample sizes. However, the present sample was already characterized by a high level of clinical impairment, indicated by an average of 2.38 current mental disorders and a high co-occurrence of suicidality, which may explain the nonsignificant differences. Further research in broader clinical samples, for example, including outpatients, is needed. Furthermore, these groups may differ regarding other characteristics e.g., injury severity or specific NSSI methods, which should also be considered when designing further studies.

The present study confirmed the findings about high co-occurrence of suicidal behavior and NSSI in a clinical sample of adolescents with NSSI-D (7). In this sample, 96.3% of the adolescents with NSSI-D reported lifetime suicidal ideation, about 70% a history of suicide attempts, and 66% current suicidal ideation, which is similar to findings of previous studies (13, 49). Moreover, 60% additionally fulfilled research criteria for SBD, with no difference between the NSSI-AF and NSSI-OF groups. Consistent with previous research (13, 14, 50) and in line with the gateway theory, our results support the idea that NSSI takes place before first suicide attempts occur. We found that NSSI and suicidal ideation start around the same age, in line with the results of Groschwitz et al. (13).

We further investigated predictors of current suicidal ideation and lifetime suicide attempts to replicate previous findings. We first investigated predictors of occurrence of current suicidal ideation and suicide attempts. Our results indicate that a higher number of NSSI methods (OR = 1.81) significantly predicts lifetime suicide attempts, which is in line with previous research (e.g., (7, 19). Consistent with findings from Andrewes et al. (51) and Burke et al. (27), higher levels of depressive symptoms significantly predict current suicidal ideation (OR = 1.09). Previous studies (27, 28) found that the anti-suicide function was an important predictor of occurrence of suicidal ideation. Our findings suggest that the anti-suicide function does not significantly predict occurrence of current suicidal ideation and lifetime suicide attempts, but this might be explained by the small sample size reporting anti-suicide functions. But it should be noted that our sample included adolescent inpatients and an in-depth clinical assessment was used. A further explanation is the high co-occurrence with suicidality as a dichotomous variable.

Moreover, Burke et al. (27) found an additive effect on the anti-suicide function in individuals with severe depressive symptoms. Because of the small sample size of individuals engaging in NSSI to avoid suicide, we could not perform further analyses. In this study, the anti-suicide function, number of NSSI methods, and NSSI frequency were not correlated with the current level of depressive symptoms, which is in line with the finding that there are stronger associations between suicidality, especially suicide attempts, and NSSI than between NSSI and depressive symptoms (52). Focusing on intensity and duration of current suicidal ideation, our results indicate that higher frequency of expecting to avoid suicide leads to longer duration of current suicidal ideation. This finding underlines the important relation between NSSI to avoid suicide and the current level of suicidal ideations, which was also reported in prior research (28). In one of the few studies on the anti-suicide function, Paul et al. (20) found that the anti-suicide function was more frequently reported in university students who had attempted suicide than those who reported lifetime suicidal ideation. Thus, the direction of the association between the anti-suicide function and duration of current suicidal ideation remains unclear. For example, engaging in NSSI to avoid suicide may also be a consequence of longer duration of suicidal ideation. Thus, individuals with current suicidal ideation may experience intense emotions and an ambivalence about the future, while spending time contemplating a suicide attempt. The possibility of engaging in NSSI to avoid suicide may represent a short-term protective factor against suicidal behavior (in line with the anti-suicide model), while thinking about the desire to die may have an impact on the duration of suicidal ideation. However, it should be noted that the design of the study is cross-sectional and therefore longitudinal studies are necessary to investigate the association between NSSI to avoid suicide and duration of current suicidal ideation. To develop effective prevention and treatments, further research is needed to clarify this relationship. Our results indicate that higher NSSI frequency in the past 6 months significantly predicts lower intensity of current suicidal ideation. Our results therefore replicate findings from Paul et al. (20) demonstrating a curvilinear association that the risk for suicidal thoughts and behaviors peaked and then declined as the number of lifetime NSSI episodes increased. Whitlock and Knox’s (53) explanation for this relationship is that frequent NSSI has become a “working,” albeit maladaptive, coping strategy to deal with distress such as suicidal ideation.

The results of the present study should be interpreted in the context of the following limitations. Our findings regarding the comparison between the anti-suicide function and other functions are based on a reduced sample size. Given the small sample size, a replication is necessary. Furthermore, the sample of the current study included only female adolescents with NSSI-D recruited from child and adolescent psychiatric units and thus the results may not be generalizable to male adolescents. Moreover, the design of the study is cross-sectional. It should be noted that the characteristics associated with suicidality and NSSI, especially NSSI functions before, during, and after NSSI, were assessed using retrospective self-report measures. According to Klonsky (25), memories about NSSI may be inaccurate, and supplementary laboratory studies, such as ecological momentary assessment (EMA), could be used to increase validity. Thus, additional longitudinal studies will be needed to clarify the relationship and causality between NSSI, the anti-suicide function, and suicidality. The strengths of the study are the assessment of NSSI-D and suicidality with a structured clinical interview according to the *DSM-5* research criteria and the assessment of different functions of NSSI in a clinical sample. In addition, investigating the association between a specific NSSI function and NSSI in adolescent inpatients is an important strength of the study.

In conclusion, this study provides preliminary support for the belief that female adolescents with NSSI frequently use NSSI to avoid suicide. In light of the high co-occurrence of NSSI and suicidality, our results underline the importance of in-depth clinical assessment of suicidality and of several NSSI functions in adolescents with NSSI. Further research is needed to clarify the relationship between NSSI and suicidality, investigating predictors for trajectories of NSSI and suicidality, for example, frequency of NSSI, number of methods, various NSSI functions—including the anti-suicide function—and the role of negative reinforcement. Therefore, longitudinal studies with large-enough sample sizes to investigate causality and relevant psychological mechanisms, including, for example, EMA methods, are needed to better understand reinforcement mechanisms in NSSI.

## Data Availability Statement

The datasets used are available from the corresponding authors on reasonable request.

## Ethics Statement

The studies involving human participants were reviewed and approved by Lokale Ethikkommission beider Basel. Written informed consent to participate in this study was provided by the participants’ legal guardian/next of kin.

## Author Contributions

LK completed the data analyses and made substantial contributions to the interpretation of the data and the drafting of the manuscript. TI-A and MS contributed to the ideas, the acquisition and interpretation of the data, and the drafting of the manuscript. All authors read and approved the final manuscript.

## Funding

This study is supported by grant project 100014_135205 awarded to TI-A in collaboration with MS by the Swiss National Science Foundation.

## Conflict of Interest

The authors declare that the research was conducted in the absence of any commercial or financial relationships that could be construed as a potential conflict of interest.
